# Interstitial round needles should not be used for cervical cancer patient treated with intracavitary/ interstitial brachytherapy using a Venezia applicator : a case report

**DOI:** 10.1186/s12905-024-03091-2

**Published:** 2024-04-18

**Authors:** Yoshiaki Takagawa, Sachiko Izumi, Eiichi Takahashi, Midori Kita

**Affiliations:** 1 Department of Minimally Invasive Surgical and Medical Oncology, Fukushima Medical University, 1 Hikarigaoka, Fukushima-shi, Fukushima, 960-1295 Japan; 2 Department of Radiation Oncology, Southern TOHOKU General Hospital, Fukushima, Japan; 3 Department of Radiology, Tokyo Metropolitan Tama Medical Center, Tokyo, Japan

**Keywords:** Cervical cancer, Interstitial brachytherapy, Venezia applicator, Uncertainty

## Abstract

**Background:**

Image-guided adaptive brachytherapy (IGABT) demonstrates an excellent local control rate and low toxicity while treating cervical cancer. For intracavitary/interstitial (IC/IS) brachytherapy (BT), several applicators are commercially available. Venezia (Elekta, Sweden), an advanced gynecological applicator, is designed for IC/IS BT for treating locally advanced cervical cancer. There are two types of interstitial needles for the Venezia applicator: the round needle and sharp needle. Generally, a round needle is safer because it has less risk of damaging the organ at risk than a sharp needle. However, there is currently no evidence to suggest that a round needle is better than a sharp needle for the Venezia applicator in IC/IS BT. Herein, we documented our experience of using both round and sharp needles with the Venezia applicator in IC/IS BT for cervical cancer.

**Case presentation:**

A 71-year-old woman was diagnosed with clinical stage T2bN0M0 and the International Federation of Gynecology and Obstetrics stage IIB cervical squamous cell carcinoma. Definitive therapy, including a high-dose-rate BT boost, was planned using a round needle with the Venezia applicator in IC/IS BT. After inserting four interstitial round needles during the first and second BT sessions, an unexpectedly large gap (1.5 cm) was detected between the cervix and ovoid. We therefore used a sharp needle with the Venezia applicator for IC/IS BT during the third and fourth BT sessions. Three sharp needles were firmly inserted during the third and fourth BT sessions.

**Conclusions:**

The study findings suggest that the interstitial round needle should not be used for cervical cancer patients undergoing IC/IS BT using the Venezia applicator.

## Background

**Fig. 1:**
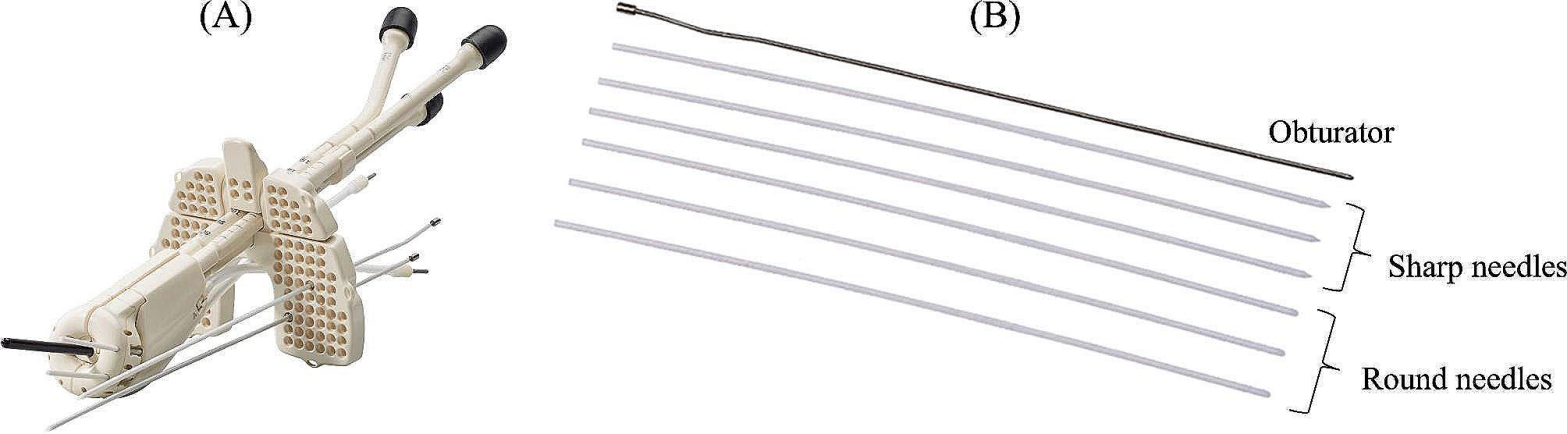
(A) Image of the Advanced Gynecological Applicator Venezia (Elekta, Sweden). (B) Image of the obturator, ProGuide sharp needle and ProGuide round needle (Elekta, Sweden)

Image-guided adaptive brachytherapy (IGABT) demonstrates an excellent local control rate and low toxicity while treating cervical cancer [1]. For intracavitary/interstitial (IC/IS) brachytherapy (BT), several applicators are commercially available [2]. Venezia (Elekta, Sweden), an advanced gynecological applicator, is designed for IC/IS BT for treating locally advanced cervical cancer (Fig. 1A). There are two types of interstitial outer needles for the Venezia applicator: the ProGuide round needle and ProGuide sharp needle (Elekta, Sweden) (Fig. 1B). An inner obturator is also required to insert the needle. A round needle is often safer than a sharp needle since it is less likely to cause injury to the organ in issue, but there is no evidence to support this for the Venezia applicator in IC/IS BT.

Herein, we documented our experience of using both round and sharp needles with the Venezia applicator in IC/IS BT for cervical cancer.

## Case presentation

**Fig. 2:**
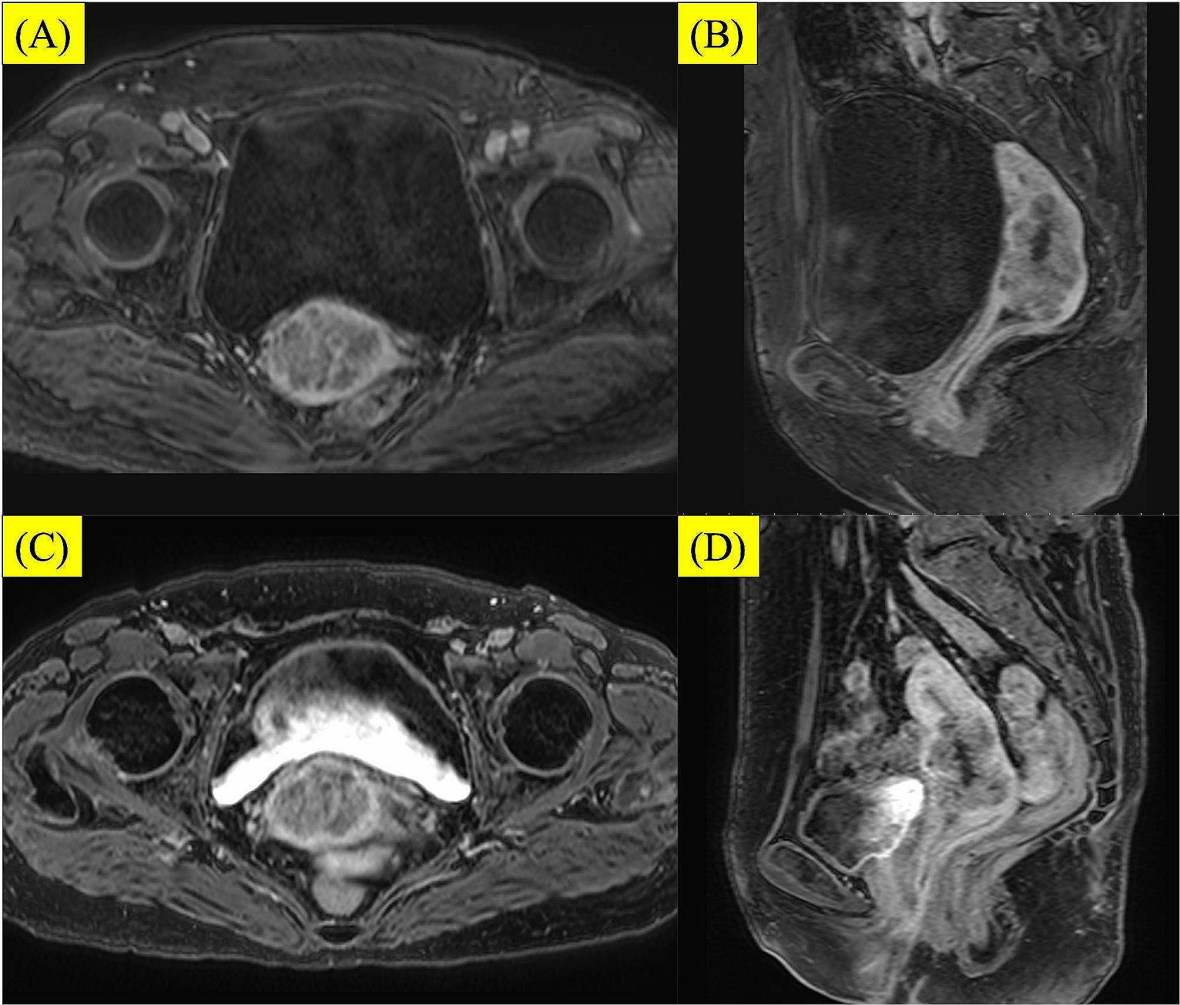
A, B) Pretreatment contrast-enhanced magnetic resonance imaging (MRI) showed a bulky tumor in the cervix. C, D) Interim MRI before brachytherapy showing no significant tumor shrinkage

A 71-year-old woman was diagnosed with clinical stage T2bN0M0 and the International Federation of Gynecology and Obstetrics stage IIB cervical squamous cell carcinoma. The primary tumor extended to the right parametrium, and the initial tumor size was 4 × 4 × 6 cm (Fig. 2A-B). At the age of 30 years, she was diagnosed with rheumatoid arthritis and had been on immunosuppressants for a prolonged period. Thus, we planned definitive chemoradiotherapy (CRT) with weekly nedaplatin and external beam radiation therapy (EBRT) followed by BT. The EBRT dose was 50.4 Gy delivered in 28 fractions to the whole pelvis. A midline block (3 cm wide at the isocenter) was inserted into the treatment field after delivering 30.6 Gy/17 fractions to the whole pelvis. After the 30.6-Gy irradiation, weekly definitive BT was introduced. Interim magnetic resonance imaging (MRI) before the first BT session showed no shrinkage of the cervical tumor (Fig. 2C-D).

### Using a round needle with the venezia applicator in IC/IS BT

**Fig. 3:**
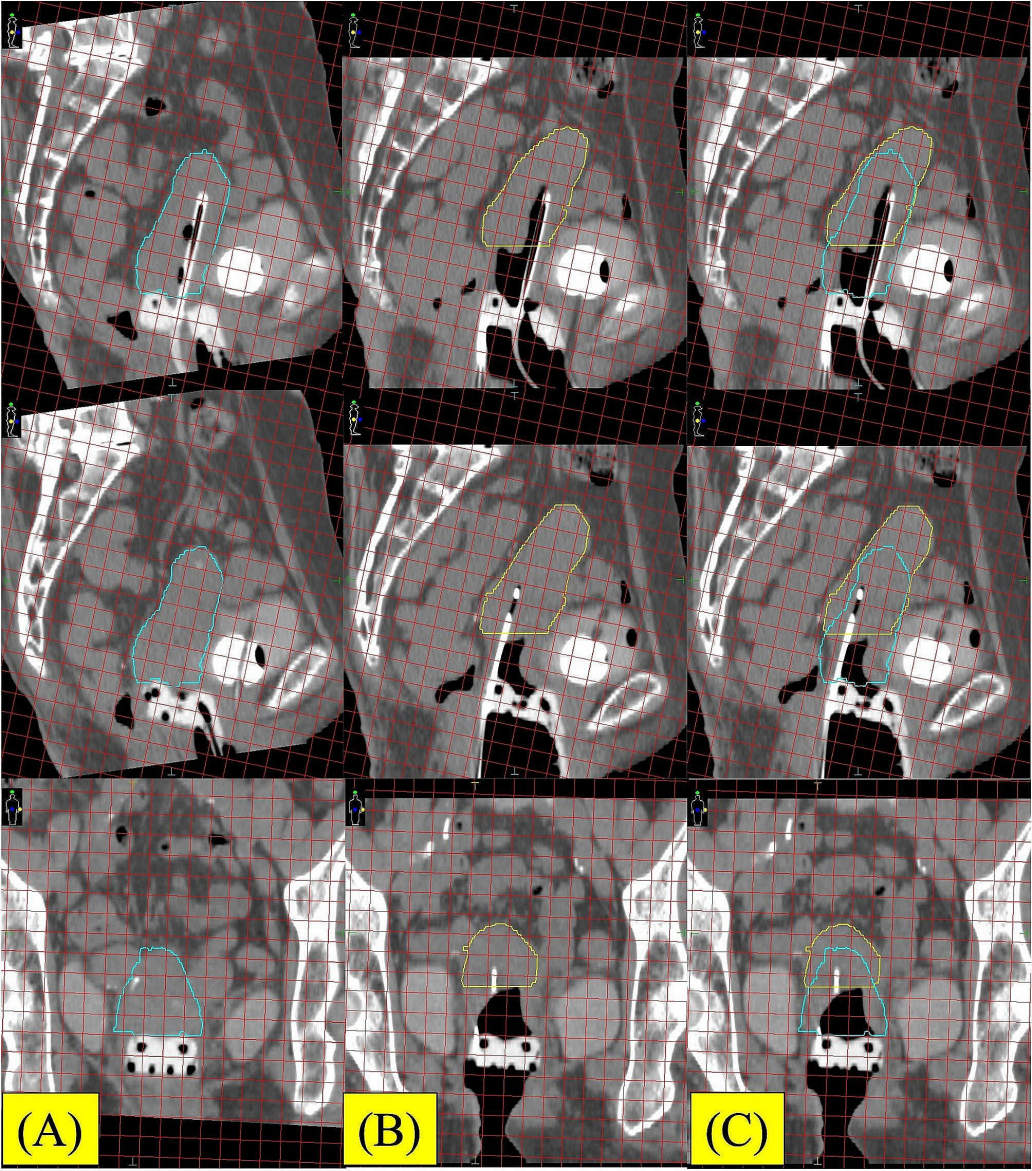
Sagittal and coronal image of the 1st BT with 1 cm grid line. (A) Before needle insertion. Right blue line; uterus. (B) After insertion of the round needles. Yellow line; uterus. (C) Image fusion with A and B. Significant uterine motion is observed after round needle insertion

**Fig. 4:**
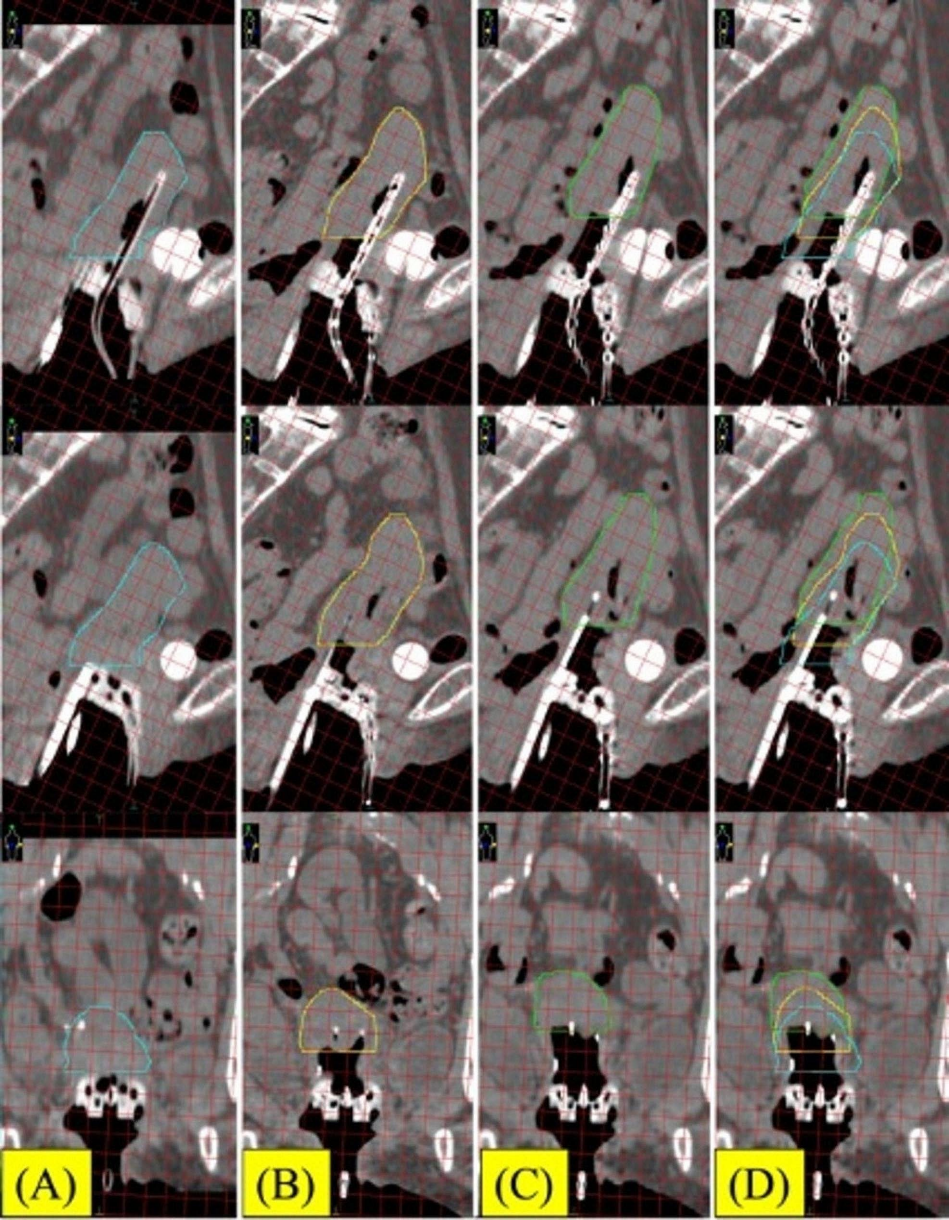
Sagittal and coronal image of the 2nd BT with 1 cm grid line. (A) Before needle insertion. Right blue line; uterus. (B) After initial insertion of the round needles. Yellow line; uterus. (C) After additional insertion of the round needles. Green line; uterus. (D) Image fusion with A - C. Significant uterine motion is observed after needle insertion

**Fig. 5:**
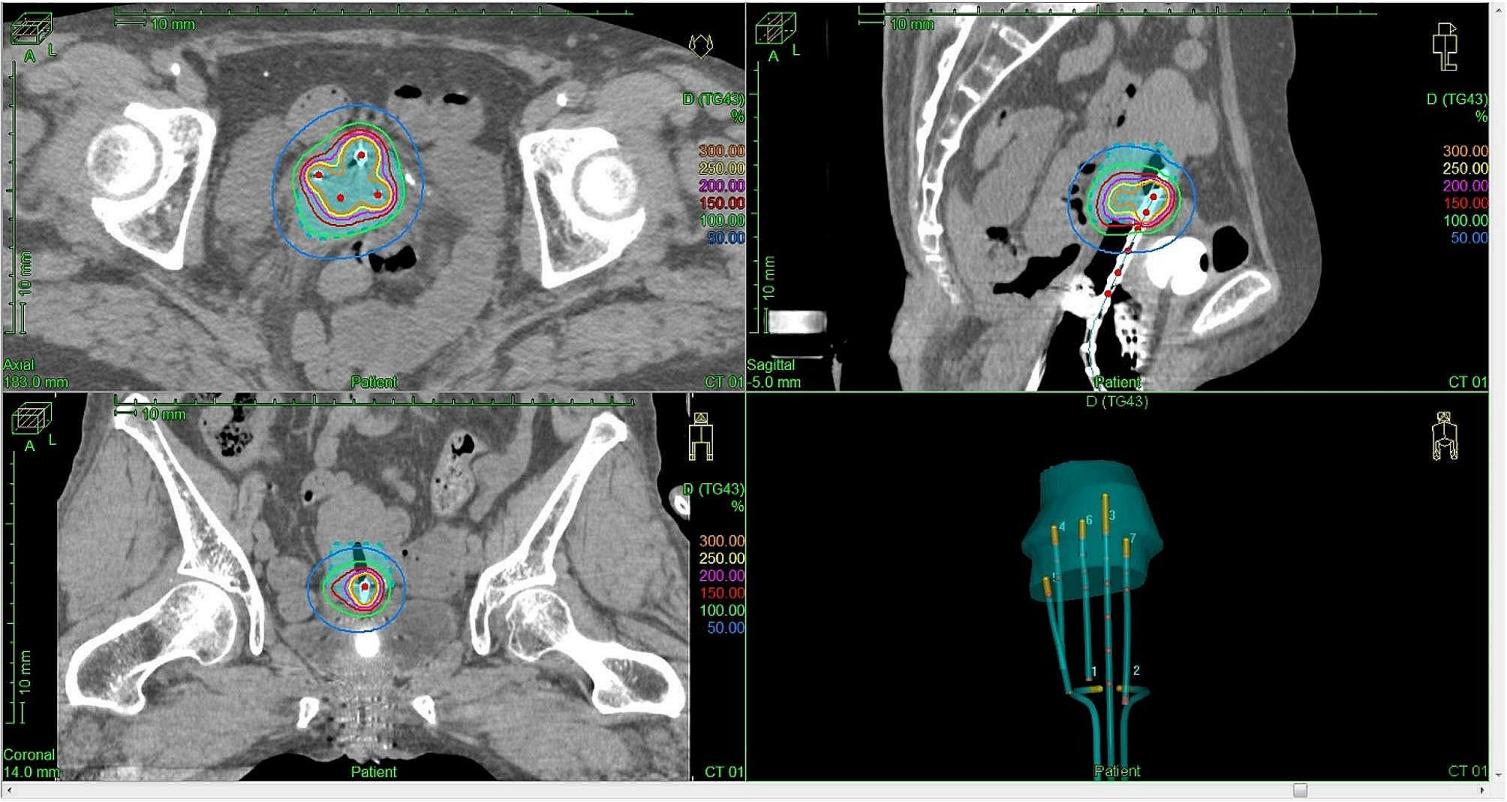
Dose distribution and applicator reconstruction of 2nd BT. Axial, sagittal and coronal view of treatment planning and 3D image of the applicator and needle reconstruction. Light blue shade; HR-CTV. Green line is 100% (6 Gy) isodose line. Blue line is 50% (3 Gy) isodose line

We planned for IC/IS BT with the Venezia applicator. The microSelectron HDR-V3 with Oncentra Brachy (Elekta, Sweden) with Ir-192 was used. The BT dose was 24 Gy in four fractions. In the first and second BT sessions, we used the ProGuide round needle (6Fr × 294 mm). In the first BT session, after placing the tandem and lunar ovoid into the patient’s uterus and vagina, we inserted four round needles (three parallel and one oblique needle) quickly into the right parametrium using an insertion tool. During applicator implantation, anesthesia was induced using an intravenous injection of midazolam, pentazocine, and hydroxyzine hydrochloride. We then performed computed tomography (CT) for treatment planning, and a large space (1.5 cm) was detected between the cervix and ovoid (Fig. 3). First, we hypothesized that this phenomenon was caused by the interstitial round needle not being inserted firmly or the lunar ovoid not being placed in the optimal position before needle insertion. The dose distribution during the first BT session was not satisfactory because of the large space between the cervix and the ovoid. In the second BT session, we performed the same procedure; however, an additional CT scan was performed just before needle insertion (Fig. 4A). Subsequently, we immediately inserted four round needles (three parallel and one oblique needle) into the right parametrium using an insertion tool. Unfortunately, CT after needle insertion again showed a large space (1.5 cm) between the cervix and ovoid (Fig. 4B). To improve dosimetric coverage for the high-risk clinical target volume (HR-CTV), we attempted to insert an additional 1 cm of the interstitial round needles. However, the gap widened by another 1 cm (Fig. 4C-D). Therefore, the dose distribution of the second BT session was worse than that of the first (Fig. 5). After the first and second BT sessions, we considered that perhaps the round needles were not being inserted firmly into the cervical tissue during IC/IS BT using the Venezia applicator.

### Using a sharp needle with the venezia applicator in IC/IS BT

**Fig. 6:**
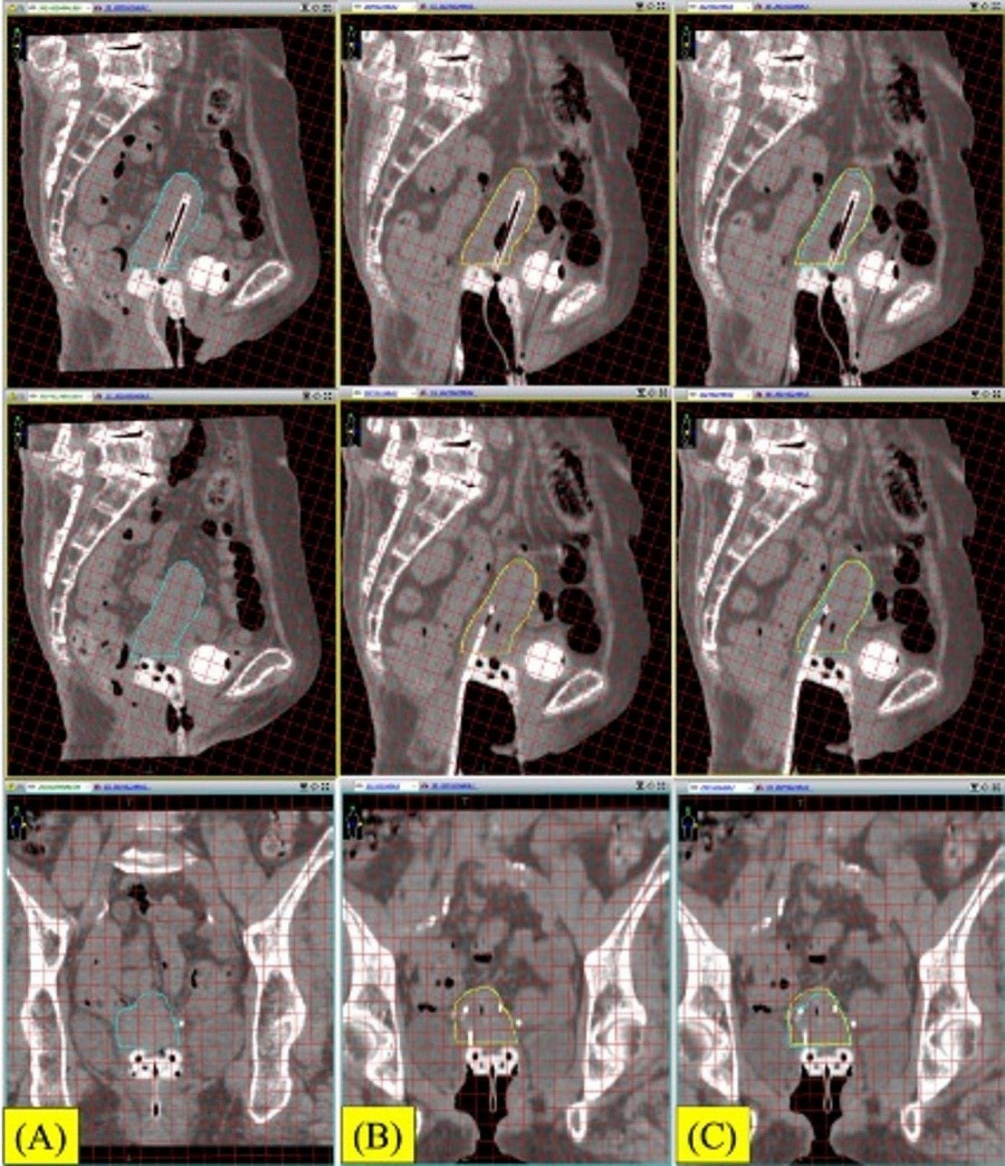
Sagittal and coronal image of the 3rd BT with 1 cm grid line. (A) Before needle insertion. Right blue line; uterus. (B) After insertion of the sharp needles. Yellow line; uterus. (C) Image fusion with A - B. Uterine motion is not observed after needle insertion

**Fig. 7:**
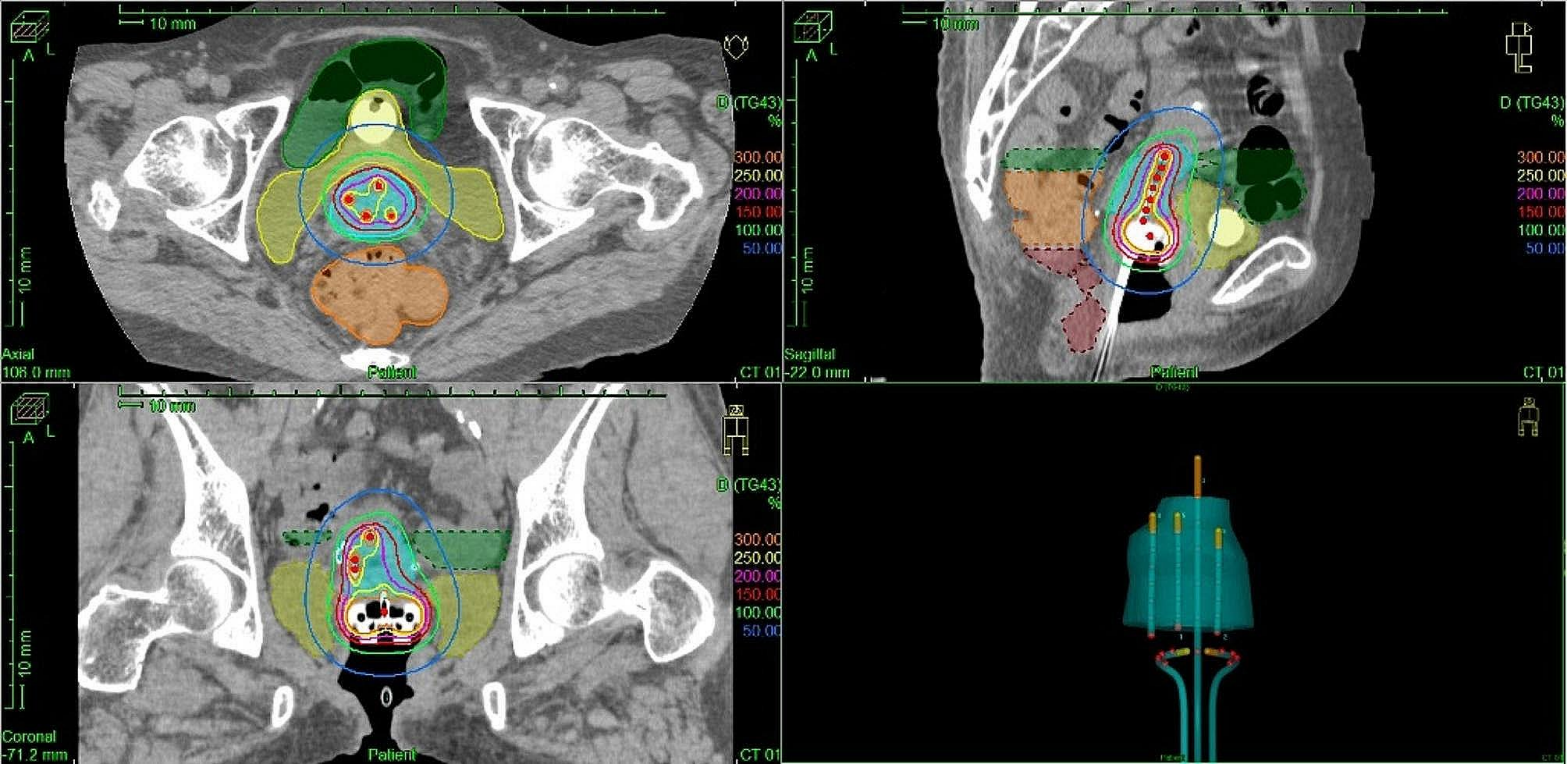
Dose distribution and applicator reconstruction of 3rd BT. Axial, sagittal and coronal view of treatment planning and 3D image of the applicator and needle reconstruction. Light blue shade; HR-CTV. Green line is 100% (6 Gy) isodose line. Red line is 150% (9 Gy) isodose line

We then used a sharp needle (6Fr × 294 mm) with the Venezia applicator in IC/IS BT during the third and fourth BT sessions; three sharp needles were firmly inserted and the dose distribution was satisfactory (Figs. 6 and 7). The dosimetric parameters of each BT are described in Table 1. The total biologically equivalent doses in 2 Gy fractions (EQD2) of EBRT (30.6 Gy/17 fractions) plus BT (24 Gy/4 fractions) based on the linear-quadratic model for HR-CTV D90 were 59.9 Gy assuming an α/β ratio of 10. The EQD2 for D2 cc of the rectum, bladder, and sigmoid were 51.3 Gy, 69.6 Gy, and 51.7 Gy, respectively, assuming an α/β ratio of 3. Unfortunately, 20 months after treatment, local recurrence in the cervix and left pelvic lymph node were detected and chemotherapy was initiated.

**Table 1:** Dosimetric parameters of each brachytherapy

Brachytherapy	HR-CTV D90 (Gy)	HR-CTV D98 (Gy)	HR-CTV V100 (%)	Rectum D2cc (Gy)	Bladder D2cc (Gy)	Sigmoid D2cc (Gy)
# 1 (round needles)	5.0	3.6	80.33	3.8	6.1	4.5
# 2 (round needles)	3.4	2.6	67.11	1.4	3.6	2.6
# 3 (sharp needles)	7.4	6.4	99.63	4.3	6.3	3.6
# 4 (sharp needles)	6.3	5.9	91.93	5.0	6.2	4.5

HR-CTV, high-risk clinical target volume; D_n_ (_90, 98_), minimal dose delivered to n% of target volume; V_100_, fractional volume of the organ receiving 100% of the prescribed dose; Rectum D_2cc_, Bladder D_2cc_ and Sigmoid D_2cc_, doses for most exposed 2 cc volumes of rectum, bladder and sigmoid, respectively;

## Discussion and conclusions

BT played an important role in treating cervical cancer during the two-dimensional (2D) era [3]. Recently, IGABT for treating locally advanced cervical cancer is being widely recognized as a new paradigm, replacing 2D BT, and its utilization has increased worldwide [4]. BT in the treatment of cervical cancer carries several uncertainties, including source calibration, dose and dose–volume histogram calculation, reconstruction of applicators, contouring, intra- and inter-fractional uncertainties, and dose delivery [5]. Several studies have been conducted on uterine intra- and inter-fractional motion during EBRT. Uterine motion is predominantly influenced by bladder filling and cervical motion via rectal filling. A systematic review reported on large population-based planning target volume margins (up to 4 cm around the uterus) [6]. Although the uterus is surrounded by several ligaments, including the cardinal ligament, these results showed that it is a significantly mobile organ.

Similar to EBRT, there are intra- and inter-fractional uncertainties regarding the uterus and applicator in BT. Several studies have reported on intra-fractional applicator displacements during each high-dose rate BT fraction [7–12]. Unfortunately, there are few reports on uterine motion during IC/IS BT. To the best of our knowledge, this is the first case report of intra-fractional uterine motion during IC/IS BT, using the Venezia applicator. Because the tip of the round needle is significantly less sharp, we considered that it may only push the tissue instead of penetrating it firmly. Therefore, interstitial round needles should be inserted into the applicator such as a multichannel cylinder, which does not need to puncture the patient’s tissue. While, based on our findings, interstitial sharp needles should be used for IC/IS BT for cervical cancer using recent hybrid applicators including the Venezia/Geneva applicator (Elekta, Sweden).

During IC/IS BT without the Venezia applicator, we always insert an interstitial needle under transrectal ultrasound guidance. However, transrectal ultrasound-guided interstitial needle insertion is difficult because of the large size of the Venezia applicator. Although transabdominal ultrasound-guided interstitial needle insertion is available, this method does not produce clear enough images for insertion. In our case, there was a risk of looseness of the uterine ligament because of her age. Moreover, the anesthetic drugs may have loosened the uterine ligament. However, a large gap between the cervix and the applicator in IC/IS BT using the Venezia applicator cause significant negative impact for the dose distribution. (Figures 3, 4 and 5; Table 1).

Due to the standard CRT including IGABT for locally advanced cervical cancer using modern imaging techniques, the 5-year local control rate was over 90% [1]. On the other hand, the 5-year disease-free survival rate was 68%. This means that although the primary tumor of the cervix is well controlled by CRT, almost one third of the patients have had lymph node recurrence or distant metastasis after initial treatment. However, in recent years, the development of pharmacotherapy including immune check point inhibitors has led to significant improvements in the outcomes of recurrent/metastatic cervical cancer [13].

In the EMBRACE-I study, the 5-year incidence of grade 3–5 morbidity was 6.8% for genitourinary events, 8.5% for gastrointestinal events, 5.7% for vaginal events, and 3.2% for fistulae [1]. Higher doses to the vagina are associated with ≥ grade 2 vaginal stenosis [14]. Recently, a novel hypothesis has been proposed that reactive oxygen species within the vaginal space may increase the risk of cervical intraepithelial neoplasia and uterine cervical cancer development, in addition to the widely known causative effect of human papillomavirus infection [15]. Therefore, more attention should be paid to the vaginal microenvironment in the future.

In conclusion, the study findings suggest that the interstitial round needle should not be used for cervical cancer patients treated with IC/IS BT using the Venezia applicator. Radiation oncologists should consider which interstitial needle is optimal for each hybrid applicator in treating cervical cancer.

## Data Availability

The dataset generated for this report is available from the corresponding author upon reasonable request.

## Abbreviations

IGABT: Image-guided adaptive brachytherapy
IC/IS BT: Intracavitary/interstitial brachytherapy
EBRT: External beam radiation therapy
MRI: Magnetic resonance imaging
CT: Computed tomography
HR-CTV: High-risk clinical target volume
EQD2: Equivalent doses in 2 Gy fractions
